# The endonasal endoscopic surgical approach to challenging pediatric frontal sinus inflammatory lesions

**DOI:** 10.1093/jscr/rjaf673

**Published:** 2025-09-06

**Authors:** Baqer A Aldhneen, Maria R Alabdulaal, Hussain J Aljubran, Abdulrahman G Alharbi, Eman R Alanazi, Ali Almomen

**Affiliations:** Department of Otolaryngology Head and Neck Surgery, Alahsa Health Cluster, Al Hofuf 36375, Saudi Arabia; Department of Otolaryngology Head and Neck Surgery, Aljabr Eye and ENT Hospital, Alahsa Health Cluster, Alahsa 36375, Saudi Arabia; Department of Otolaryngology Head and Neck Surgery, Aljabr Eye and ENT Hospital, Alahsa Health Cluster, Alahsa 36375, Saudi Arabia; Department of Otolaryngology Head and Neck Surgery, King Fahad Specialist Hospital, Dammam 32253, Saudi Arabia; Department of Otolaryngology Head and Neck Surgery, Maternity And Children Hospital, Dammam 32253, Saudi Arabia; Department of Otolaryngology Head and Neck Surgery, King Fahad Specialist Hospital, Dammam 32253, Saudi Arabia

**Keywords:** pediatric frontal sinus, endoscopic sinus surgery, mucocele, mucopyocele, allergic fungal sinusitis

## Abstract

A variety of pathologies and anatomical variations contribute to the underreporting of pediatric paranasal sinus disorders. The frontal sinus presents significant risk for complications due to its proximity to the orbit and brain. Three cases encountered in a tertiary hospital are discussed in this study to illustrate the usefulness of endonasal endoscopic methods in addressing pediatric frontal sinus lesions. These included bilateral frontal sinus mucopyoceles, bilateral frontal allergic fungal sinusitis, and a unilateral frontal mucocele, all of which were completely resolved with no recurrence during follow-up. Diagnosis is often delayed in pediatric patients due to their unusual presentations. In conclusion, endoscopic sinus surgery is the cornerstone for managing challenging pediatric frontal sinus disease, showing good recovery and superior cosmetic outcomes. The minimally invasive endoscopic approach is recommended for these conditions.

## Introduction

Paranasal sinuses are air-filled cavities in the skull located around the nasal cavity. At birth, the maxillary and ethmoid sinuses are present and typically fully develop by the age of three. The sphenoidal sinus begins developing around age three, and the frontal sinus starts around age seven, with full development occurring during adolescence [1].

Paranasal sinus diseases in the pediatric population are not well documented. This is due to various factors, including anatomical variability by age, differences in sinus development patterns, and a wide range of pathologies, including congenital malformations, inflammatory diseases, trauma, and neoplasms [2]. The frontal sinus carries significant risks for complications due to its proximity to the orbit and brain [3]. This case series aims to highlight the value of the endonasal endoscopic approach in managing various frontal sinus inflammatory pathologies in the pediatric population.

## Case series

### Case 1. Bilateral frontal sinus mucopyoceles in a pediatric patient

This is a 17-year-old male, known case of type 1 insulin-dependent diabetes mellitus and a history of functional endoscopic sinus surgery (ESS) performed one year earlier at another tertiary center. He presented to our otolaryngology clinic complaining of bilateral nasal obstruction, anosmia, epistaxis, facial heaviness, and a left-sided headache. Nasal endoscopy revealed bilateral grade 4 nasal polyps with white nasal discharge. Ocular examination showed left eye proptosis with displacement downwards and outwards.

**Figure 1:**
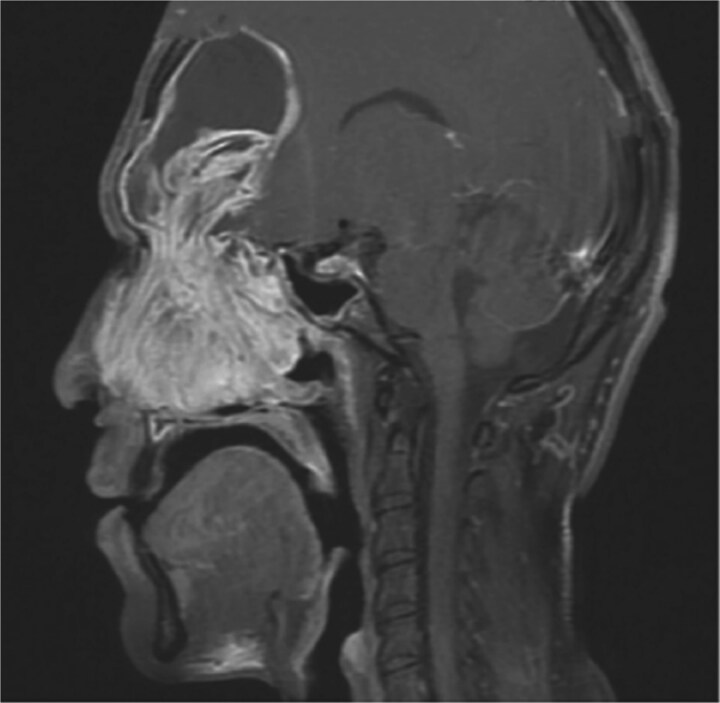
MRI sagittal view of the brain and sinuses showing mucopyoceles causing significant bilateral frontal sinus enlargement.

Preoperative non-contrast computed tomography (CT) scan of paranasal sinuses showed bilateral frontal sinus expansion, more pronounced on the left side with posterior wall dehiscence and soft tissue extension intracranially within the epidural space, measuring 5.3 × 4.1 cm. Preoperative MRI confirmed bilateral frontal sinus enlargement, with the left lesion measuring 4.7 × 6.5 × 6.7 cm and displacing the dura (Fig. 1).

**Figure 2:**
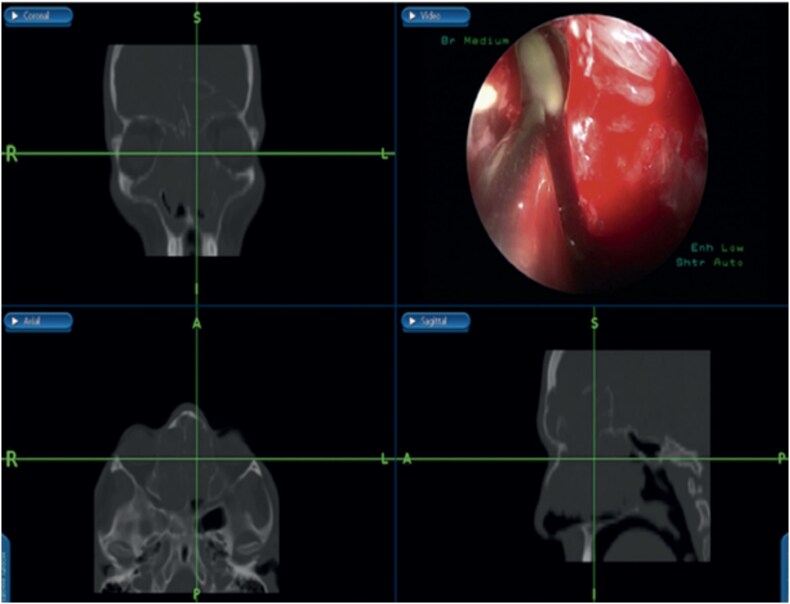
Bilateral frontal sinus showing infected nasal polyps, identified intraoperatively, and drained by image-guided endoscopic sinus surgery.

Bilateral extensive nasal polyposis was removed, and pus was drained from the ethmoid sinus cavities using a microdebrider (Fig. 2). Draf type IIb procedure was performed bilaterally to facilitate frontal sinus drainage and ventilation. The procedure was followed by antibiotic-soaked irrigation and evacuation of both mucopyocele cavities. After 6 months, the patient came to the clinic for follow-up, and his repeated CT scans showed normal sinus aeration and complete resolution.

### Case 2. Bilateral frontal allergic fungal sinusitis in a pediatric patient

**Figure 3:**
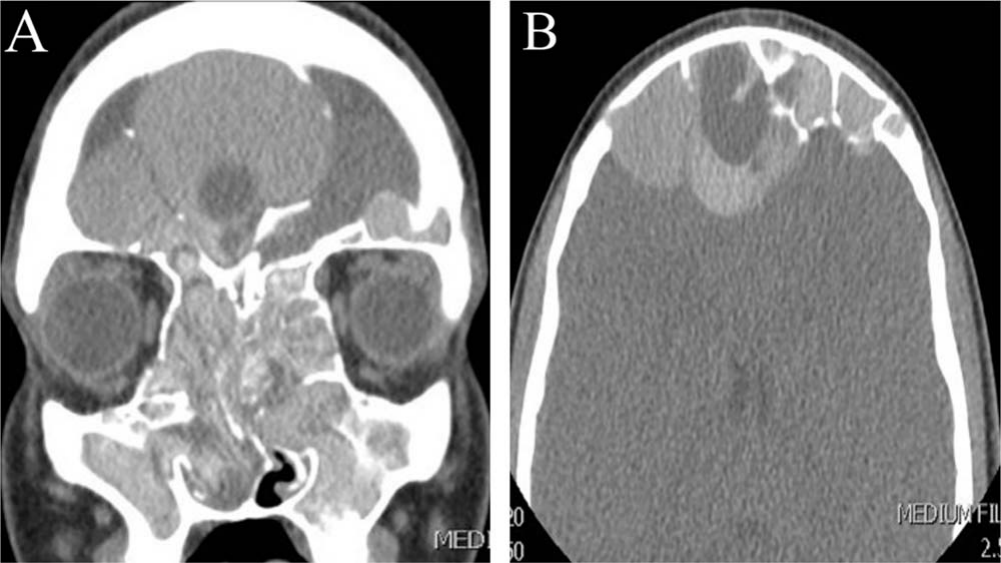
CT scan of paranasal sinuses in (A) coronal and (B) axial views, showing extensive disease with enlargement of sinuses, with more expansion of the right frontal sinus.

This is a 15-year-old male with no significant medical or surgical history who presented to our otolaryngology clinic with nasal discharge, mild headaches, and nasal itchiness. Preoperative CT scan of the paranasal sinus showed extensive disease with enlargement of the sinuses, predominantly on the right side (Fig. 3). MRI of the brain and paranasal sinus revealed similar findings, with right frontal sinus enlargement and intracranial extension, but intact dura.

The patient underwent image-guided ESS. Thick allergic mucin was aspirated and removed from the sinuses, including near the pulsating dura, with no cerebrospinal fluid leak observed during the surgery. Postoperatively, he received a short course of systemic corticosteroids (oral prednisone taper over 3 weeks), intranasal steroids, and a 7-day course of antibiotics to prevent secondary infection. At three-year follow-up, CT scans confirmed no recurrence.

### Case 3. Unilateral frontal mucocele in a pediatric patient

This is a 14-year-old female, with no significant medical or surgical history, who presented to our otolaryngology clinic complaining of right frontal bone depression associated with headache for one month, with no other ENT-related symptoms. Physical examination and nasal endoscopy were unremarkable.

**Figure 4:**
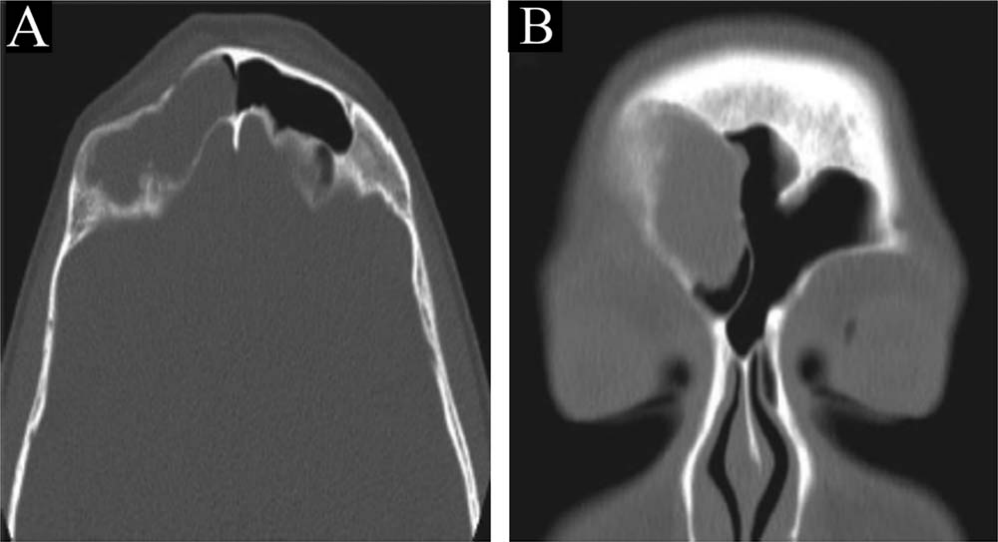
CT scan of the brain and paranasal sinuses in (A) axial and (B) coronal views, showing a lesion in the right frontal sinus with resorption of posterior and lateral walls. The anterior wall shows a depressed fracture.

A CT scan of the paranasal sinuses showed a low-attenuation lesion in the right frontal sinus lateral aspect with thinning and resorption of the sinus walls and depression in the posterior side of the anterior wall, suggestive of a depressed fracture (Fig. 4). MRI of the brain and paranasal sinuses showed no thickening of the meninges and no intracranial extension.

**Figure 5:**
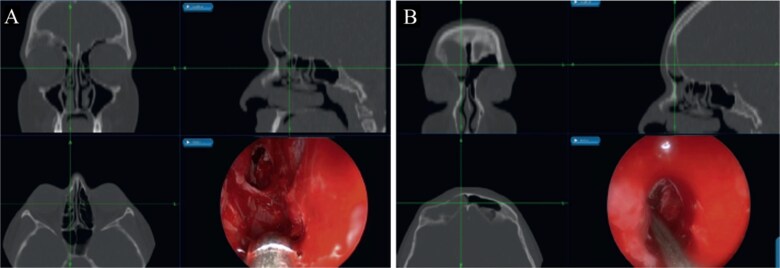
(A) Mucocele Draf IIa wide frontal sinusotomy, (B) image-guided wide marsupialization of mucocele.

The patient underwent image-guided ESS with frontal sinusotomy. A 45-angled endoscope helped in recognition of the mucocele, which was treated with marsupialization (Fig. 5). The thick anterior inferior wall was removed. During 2 years of follow-up, no recurrence or complication was observed.

## Discussion

Pediatric paranasal sinuses can be affected by a wide spectrum of pathologies. Most of these are self-limiting conditions, but some may become complicated and potentially life-threatening [4, 5]. These cases illustrate a spectrum of frontal sinus pathologies, such as mucopyoceles, mucoceles, and allergic fungal rhinosinusitis (AFRS). In all three cases, the primary pathologies were either inflammatory lesions (AFRS) or obstructive lesions (mucoceles and mucopyoceles), managed endoscopically in a tertiary hospital setting.

Pediatric frontal sinus inflammatory diseases are relatively rare but can be extremely aggressive, with up to 30% of cases resulting in orbital or intracranial complications due to their proximity to major structures such as the dura and orbit [6]. Diagnosing complicated sinusitis in pediatric patients is often delayed and difficult due to atypical presentations and a lack of nasal symptoms at disease onset [7–9]. Surgical management becomes the primary treatment in cases of complicated frontal sinusitis. AFRS in children is generally more serious than in adults, with bone erosion documented in 40%–50% of cases and proptosis in 25% [10, 11]. A retrospective analysis of 50 pediatric AFRS patients revealed a roughly 35% recurrence rate within two years after the surgery [12]. In case 2, early intracranial extension emphasizes the necessity of prompt recognition and intervention to avoid such complications. The literature suggests that postoperative corticosteroid regimens decrease recurrence rates considerably, with some studies reporting reductions ranging from 40% to 15% [13].

Paranasal mucopyoceles, while benign, can cause severe morbidity due to their expansive nature and potential for orbital or intracranial involvement. Intracranial extension is reported in 10%–15% of frontal mucopyoceles, highlighting the importance of early surgical intervention [14]. ESS has emerged as the preferred therapy for such lesions, providing better drainage and ventilation while reducing morbidity and maintaining cosmesis [15, 16]. Case 1 revealed effective therapy of bilateral frontal mucopyoceles with intracranial extension utilizing the Draf IIb surgery, which is consistent with reports claiming a 95% resolution rate with this method [17].

Endoscopic techniques minimize hospital stay by 30%–50%, shorten recovery time, and have fewer drawbacks, including CSF fluid leaks (reported at <5% in large series) [18, 19]. Our series’ long-term follow-up revealed no significant postoperative complications or recurrences, confirming the method’s safety and effectiveness.

ESS has become the favored approach for treating frontal sinus illness due to its minimally invasive nature and excellent visibility [12]. In comparison to external methods shown in Table 1, ESS has notable benefits for pediatric patients, such as lower morbidity, shorter hospital stays, fewer complications, and superior esthetic outcomes [13, 14]. External methods, such as osteoplastic flaps, were previously used but are currently reserved for extremely rare cases when endoscopic access is inadequate [15]. In comparison to external methods, endoscopic surgery minimizes hospital stays by around 40%, lowers complication rates to less than 5%, and enables faster postoperative recovery, according to a comparative analysis [16, 17].

**Table 1:** Comparison of endoscopic and external surgical approaches for pediatric frontal sinus pathology

**Parameter**	**Endoscopic approach**	**External approach**
Invasiveness	Minimally invasive	Highly invasive
Hospital stay	Shorter (1–3 days)	Longer (5–7 days)
Cosmetic outcome	No external scar	Potential facial scarring
Complication rate	<5%	10%–20%
Recovery time	Faster (1–2 weeks)	Slower (3–6 weeks)
Postoperative pain	Minimal	Moderate to severe
Indication for use	Most frontal sinus diseases	Limited to extensive disease

These findings highlight the fundamental value of endoscopic sinus surgery in treating children’s frontal sinus diseases. A growing body of data points to this method as the gold standard for treating complicated frontal sinus illness in children [20].

## Conclusion

For pediatric frontal sinus diseases such as mucopyoceles, mucoceles, and allergic fungal sinusitis, endoscopic sinus surgery is an effective as well as safe therapeutic intervention. It is often superior to external techniques due to its less invasive nature, lower morbidity, and better cosmetic results. Our case series demonstrates favorable outcomes with no major complications or recurrences. According to these outcomes, ESS can be used as a first-line therapy for children with challenging frontal sinus illness. For optimum results and recurrence prevention, interdisciplinary treatment and organized long-term follow-up protocols are crucial. Larger cohorts and comparative studies should be the main focus of future research in order to further develop standardized treatment algorithms for pediatrics.

## Data Availability

All data provided in this study are available upon request from the corresponding author.
